# Genomic and Proteomic Study of *Andreprevotia ripae* Isolated from an Anthill Reveals an Extensive Repertoire of Chitinolytic Enzymes

**DOI:** 10.1021/acs.jproteome.1c00358

**Published:** 2021-06-30

**Authors:** Silje B. Lorentzen, Magnus Ø. Arntzen, Thomas Hahn, Tina R. Tuveng, Morten Sørlie, Susanne Zibek, Gustav Vaaje-Kolstad, Vincent G. H. Eijsink

**Affiliations:** † Faculty of Chemistry, Biotechnology, and Food Science, NMBU − Norwegian University of Life Sciences, N-1433 Ås, Norway; ‡ Fraunhofer Institute for Interfacial Engineering and Biotechnology IGB, Nobelstraße 12, 70569 Stuttgart, Germany

**Keywords:** proteomics, genome analysis, chitinolytic machineries, carbohydrate-binding module, CBM, chitin, Chitinase, GH18, GH19, LPMO

## Abstract

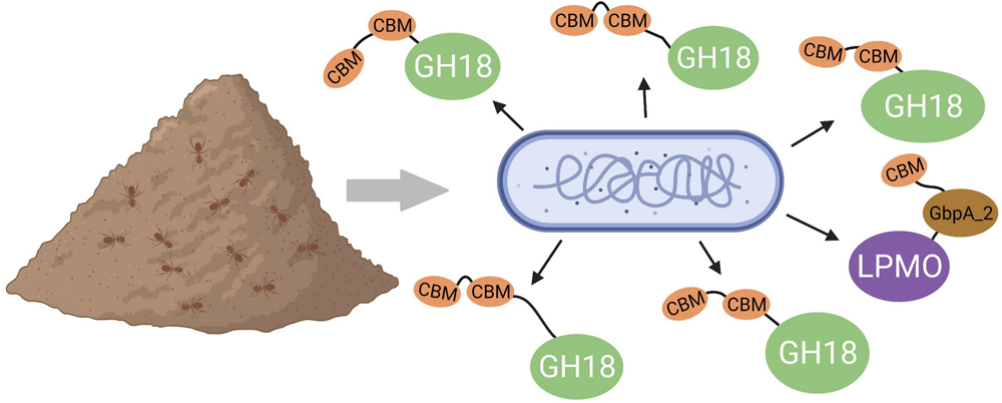

Chitin is an abundant natural polysaccharide that is hard to degrade because of its crystalline nature and because it is embedded in robust co-polymeric materials containing other polysaccharides, proteins, and minerals. Thus, it is of interest to study the enzymatic machineries of specialized microbes found in chitin-rich environments. We describe a genomic and proteomic analysis of *Andreprevotia ripae*, a chitinolytic Gram-negative bacterium isolated from an anthill. The genome of *A. ripae* encodes four secreted family GH19 chitinases of which two were detected and upregulated during growth on chitin. In addition, the genome encodes as many as 25 secreted GH18 chitinases, of which 17 were detected and 12 were upregulated during growth on chitin. Finally, the single lytic polysaccharide monooxygenase (LPMO) was strongly upregulated during growth on chitin. Whereas 66% of the 29 secreted chitinases contained two carbohydrate-binding modules (CBMs), this fraction was 93% (13 out of 14) for the upregulated chitinases, suggesting an important role for these CBMs. Next to an unprecedented multiplicity of upregulated chitinases, this study reveals several chitin-induced proteins that contain chitin-binding CBMs but lack a known catalytic function. These proteins are interesting targets for discovery of enzymes used by nature to convert chitin-rich biomass. The MS proteomic data have been deposited in the PRIDE database with accession number PXD025087.

## Introduction

Chitin is a recalcitrant linear polysaccharide comprised of β(1 → 4) linked *N*-acetyl glucosamine and is considered as the second most abundant polymer in nature, after cellulose. In nature, chitin is primarily found in the exoskeletons of crustaceans and insects and in fungal cell walls. The interest in processing of chitin to produce chitosan and chito-oligosaccharides has increased, as these chitin-derived materials have a broad range of applications in the cosmetics industry, in medicine, and as antimicrobial agents.^1−4^ Today, extraction and further processing of chitin to chitosan are done by using harsh, environmentally unfriendly chemicals.^2,5^ Since more environmentally friendly approaches toward chitin processing are desirable, there is considerable interest in the development of enzymatic methods and the discovery of novel chitin-degrading enzymes. Furthermore, the study of chitin-degrading enzyme systems may provide general insights into how nature degrades recalcitrant, insoluble polysaccharides.^6^

Many microorganisms are known to degrade chitin, as exemplified by the well-studied Gram-negative bacterium *Serratia marcescens*, that produces an efficient chitinolytic machinery.^7−9^ The chitinolytic machinery of *S. marcescens* is one of the best-known enzymatic systems for conversion of insoluble polysaccharides.^6,9^ This bacterium produces six enzymes that are involved in chitin conversion.^8,10^ These six enzymes include four chitinases belonging to glycoside hydrolase family 18 (GH18; www.cazy.org).^11^*Sm*GH18A and *Sm*GH18B are exo-acting processive enzymes, whereas *Sm*GH18C is an endo-acting non processive enzyme, and all these three enzymes have well established roles in chitin conversion.^9,12^ The role of the fourth GH18 Chitinase, *Sm*GH18D remains unclear; it is expressed at low levels during chitin degradation, has low activity on chitin, and does not improve the chitinolytic performance of a cocktail of *Sm*GH18A, B, and C.^8^ The other two enzymes are a GH20 hexosaminidase (“chitobiase”) and a lytic polysaccharide monooxygenase (LPMO), known as CBP21, which uses oxidative chemistry to cleave glycosidic bonds in crystalline chitin.^9,13,14^

While there is considerable knowledge on chitinolytic enzymes from *S. marcescens* and several other bacteria such as *Bacillus circulans*(15,16) and *Cellvibrio japonicus*,^17,18^ only a few studies have addressed the secretomes of chitinolytic bacteria growing on chitin.^8,17−20^ Such knowledge can reveal which of multiple secreted chitinases are most abundant and important during chitin turnover and may also reveal additional, hitherto unknown enzymes involved in this process. Of note, such novel enzymes could act on the chitin polymer itself but could also be involved in converting other components of the copolymeric structures that natural chitin usually is a part of, such as other carbohydrates and proteins. Published secretome studies have indeed revealed a number of “unknown” proteins potentially involved in the conversion of chitin-rich biomass (e.g., ref (17)). Since it is conceivable that bacteria isolated from chitin-enriched ecological niches contain efficient chitinolytic machineries, we have carried out an in-depth genomic and proteomic study of one such bacterium.

In search of novel and potentially more advanced chitinolytic machineries, we turned to *Andreprevotia ripae* (*A. ripae*), a Gram-negative bacterium that was isolated from an abandoned anthill.^21^ Anthills are rich in chitin, which is present in insect remains and fungi that accumulate inside the hill. We have carried out a detailed analysis of the predicted chitinolytic machinery encoded by the genome of *A. ripae*,^21^ showing the presence of a large number of carbohydrate-active enzymes (CAZymes), including an unprecedented high number of putative chitinases. We then assessed how *A. ripae* employs this rich arsenal of enzymes, by studying the secretome of *A. ripae* when grown on chitin, compared to when grown on *N*-acetylglucosamine or glucose. To do so, we used a method based on bacterial growth on agar plates rather than a method based on liquid cultures, as the former method has proven to be more effective for enriching secreted proteins, albeit not in all cases.^8,17,22^ The results show that *A. ripae* uses a huge amount of different chitinases to degrade chitin and reveals multiple proteins of unknown function that likely are involved in the degradation of chitin-rich biomass.

## Materials and Methods

### Strain and Media

*A. ripae* IGB-42^21^ was grown on M9 minimal medium plates containing 1% (w/v) milled α-chitin (Seagarden, Husøyvegen 278, 4262 Avaldsnes, Norway), 1% (w/v) glucose (VWR International, Radnor, Pennsylvania, PA, USA), or 1% (w/v) *N*-acetylglucosamine (NAG) (VWR International, Radnor, Pennsylvania, PA, USA) as sole carbon source. The plates were incubated at 22 °C, with three biological replicates for each time point and substrate. The M9 minimal medium was supplemented with 1 mM MgSO_4_ and 0.1 mM CaCl_2_, and 1% (w/v) agarose. The plates were prepared according to Bengtsson^22^ with the exception that we used glass Petri dishes with a diameter of 80 mm; hence the volume of medium per plate was reduced from 20 to 16 mL. The plates comprise two layers of identically composed solid medium (8 mL per layer) with a sterile Supor 200, 0.2 μm membrane with a diameter of 47 mm (Pall Life Sciences, Port Washington, NY, USA) placed in between the layers, in the middle of the plate. The filter separates cells (growing on the top of the plate after inoculation in the center of the plate) from the bottom of the plate, see Figure S1. The filter prevents bacteria from reaching the bottom of the plate, whereas secreted proteins migrate through the filter.^22^ During incubation of the plates, the degree to which proteins, and eventually cells, reach the lower layer under the filter will obviously change over time.

Multiple plates were inoculated by spreading out 1% of the total plate volume (160 μL) of a preculture in M9 medium with 1% glucose as the sole carbon source. When inoculating the plates, the preculture had an OD of about 0.11. For each substrate, three plates (i.e., biological replicates) were processed after 1, 3, 5, 7, and 13 days. The processing entailed that the plates were turned upside down and the agar was flipped out of the Petri dish, exposing the agar between the bottom of the Petri dish and the membrane. Using the back end of a sterile 200 μL pipet, a disc of agarose was punched out against the center of the membrane. With a layer thickness of 1.5 mm, the volume of the agarose is 30–35 μL. The gel discs were stored at −20 °C until sample preparation.

### Sample Preparation

Protein samples were prepared essentially as described by Bengtsson et al.^22^ 35 μL of 10% SDS/20 mM DTT/100 mM Tris-HCl pH 7.9 was added to each agar disc, and the sample was then incubated at 95 °C for 10 min to dissolve the agarose. The melted agarose was vortexed vigorously, and after cooling to room temperature the sample was centrifuged for 10 min at 5000 × *g* through a Ultrafree DA assembly filter (Merck Millipore, Burlington, MA, USA). The sample volume was reduced to 10–15 μL using a vacuum concentrator, and an equal amount (10–15 μL) of 2× Nu-Page buffer was added. The proteins were subjected to SDS-PAGE at 270 mV for 2 min only, using a Mini-Protean TGX Stain-free Protein gel (Bio-Rad Laboratories, Hercules, CA), and TGS as running buffer (Invitrogen, Carlsbad, CA, USA). Proteins were stained with Coomassie blue (Thermo Fisher Scientific, Waltham, MA, USA). Of note, this method does not allow determination of the protein concentration; adequate between-sample normalization was achieved during data analysis, using the MaxLFQ^23^ algorithm embedded in MaxQuant,^24^ as described below.

The protein band was cut out from the gel and transferred to Eppendorf LoBind tubes (Sigma-Aldrich, Saint-Louis, MI, USA) and washed with Milli-Q water for 15 min at room temperature and 800 rpm shaking; the washing step was repeated twice. Decoloring was performed twice, at room temperature, by incubating the gel pieces with 50% acetonitrile/25 mM ammonium bicarbonate for 15 min at 800 rpm. The decoloring liquid was removed, followed by an incubation for 5 min in 100% acetonitrile at 800 rpm. After air drying of the gel pieces for 1–2 min, the proteins were reduced by incubating in 50 μL of 10 mM dithiothreitol/100 mM ammonium bicarbonate at 56 °C for 30 min. After incubation, the reduction solution was cooled and excess liquid was removed, after which 50 μL of 55 mM iodoacetamide/100 mM ammonium bicarbonate was added for alkylation, followed by incubation at room temperature in the dark for 30 min. Excess alkylation solution was removed and 200 μL of 100% acetonitrile was added to the gel pieces, followed by incubation for 15 min at room temperature. After air drying of the gel pieces, proteins were digested overnight with 40 ng trypsin (Promega, Mannheim, Germany) in 40 μL of 25 mM ammonium bicarbonate at 37 °C as previously described.^25^ After trypsination, the samples were cooled and spun down, and the supernatants were dried under a vacuum (Concentrator Plus, Eppendorf, Denmark), after which the peptides were dissolved in 10–15 μL 0.5% (v/v) trifluoroacetic acid, desalted using a STAGE-TIP protocol,^26^ dried again, and dissolved in 10 μL 0.5% (v/v) trifluoroacetic acid. The tryptic peptides were analyzed by liquid chromatography combined with mass spectrometry (LC-MS/MS; 5 μL per injection) as described below.

### LC-MS/MS Analysis of Tryptic Peptides

Mass spectrometry analysis was performed essentially as described by Tuveng et al.^17^ In short, peptides were analyzed using a Dionex Ultimate 3000 nanoLC-MS/MS system (Dionex, Sunnyvale, CA, USA) connected to a Q-Exactive hybrid quadrupole-Orbitrap mass spectrometer (Thermo Scientific, Bremen, Germany) equipped with a nanoelectrospray ion source. The peptides (5 μL per injection) were loaded onto a trap column (Acclaim PepMap 100, C_18_, 5 μm, 100 Å, 300 μm i.d. × 5 mm, Thermo Scientific, Bremen, Germany) and backflushed onto an analytical column (Acclaim PepMap RCLS, C_18_, 3 μm, 100 Å, 75 μm i.d. × 50 cm, Thermo Scientific, Bremen, Germany). The flow rate was 300 nL/min and the solvent gradient was 4–10% B in 2 min, to 36% B in 47 min, to 44% B in 8 min and followed by a further increase to 72% B for column washing. Solvent A was 0.1% (v/v) formic acid and solvent B was 100% (v/v) acetonitrile, 0.1% (v/v) formic acid. The Q-Exactive mass spectrometer was operated in data-dependent mode acquiring one full scan (400–1500 *m*/*z*) at *R* = 70 000 followed by (up to) 10 dependent MS/MS scans at *R* = 35 000.

### Bioinformatics for Genome and Proteome Analysis

The predicted proteome of *A. ripae* was functionally annotated using the InterProScan software.^27^ (Galaxy version 5.0.0) at the EU Galaxy server^28^ (http://usegalaxy.eu) with the databases Pfam and InterPro. Verification and annotation of CAZymes and carbohydrate-binding domains (CBMs) according to the CAZy classification^11^ were performed with dbCAN 2.0 (http://bcb.unl.edu/dbCAN2/) using version 7 of the dbCAN Hidden-Markov models.^29^ Proteins were considered putatively chitinolytic if they were predicted to belong to glycoside hydrolase (GH) families 18, 19, or 20, or to auxiliary activity (AA) family 10 (i.e., bacterial LPMOs). More details are provided in the Results and Discussion section.

MS Raw files resulting from proteome analysis were analyzed using MaxQuant^24^ version 1.6.3.3, and proteins were identified and quantified using the MaxLFQ algorithm.^23^ Samples were searched against the predicted proteome of *A. ripae* (4257 sequences) supplemented with common contaminants such as human keratin and bovine serum albumin. In addition, reversed sequences of all protein entries were concatenated to the database for estimation of false discovery rates. The tolerance level for matching the database was 6 ppm for MS1 and 20 ppm for MS/MS. Trypsin was used as digestion enzyme, and two missed cleavages were allowed. Carbamidomethylation of cysteines was used as fixed modification, whereas variable modifications included protein N-terminal acetylation, oxidation of methionines, deamination of asparagines and glutamines, and formation of pyro-glutamic acid at N-terminal glutamines. The feature “Match between runs” in MaxQuant, which enables identification transfer between samples based on accurate mass and retention time, was applied with default settings.^23^ All identifications were filtered in order to achieve a protein false discovery rate of 1%. The results from MaxQuant were further processed using Perseus (version 1.6.1.1) The data was reduced by removing proteins categorized as “only identified by site”, “reverse”, or “contaminant”. As an additional cutoff criterion, proteins were only considered present if they were detected in at least two of three replicates for at least one substrate. The LFQ intensities were log2 transformed prior to analysis. Hierarchal clustering and heat map generation were done with Euclidian distance measure and average linkage.

To predict the subcellular location of the proteins we used a combination of two prediction algorithms: The SignalP server^30^ version 4.0 (http://www.cbs.dtu.dk/services/SignalP-4.0/) with default settings for Gram-negative bacteria to predict signal peptide cleavage sites, and PRED-TAT (http://www.compgen.org/tools/PRED-TAT) to predict proteins with twin-arginine signal peptides. A protein was considered secreted if one of these two algorithms predicted it. In addition, LipoP^31^ version 1.0 (http://www.cbs.dtu.dk/services/LipoP/) was used for further annotation to separate between secreted and lipo-proteins, i.e., the presence of signal peptides cleaved by signal peptidase I (SpI) or signal peptidase II (SpII), respectively, and for prediction of cytosolic proteins (CYT).

## Results and Discussion

### Genome Analysis and the Predicted Chitinolytic Machinery of *A. ripae*

The genome of *A. ripae* was assembled from 52 contigs leading to a genome size of 4.7 Mb and a G+C content of 61.3%.^21^ Gene annotation using Prokka version 1.14.1 yielded 4257 potential open reading frames, of which 587 (13.8%) were predicted to encode for secreted proteins. A search with dbCAN^29^ showed that the genome encodes for 188 putative carbohydrate-active enzymes (4.4%), including 74 glycosyl hydrolases (GHs), 45 glycosyl transferases (GT), 29 carbohydrate esterases (CE), 3 polysaccharide lyases (PL), one lytic polysaccharide monooxygenase (LPMO), 9 proteins with other auxiliary activities (AA), and 27 proteins containing a carbohydrate-binding module (CBM) but with no predicted carbohydrate-active catalytic function.

**Figure 1:**
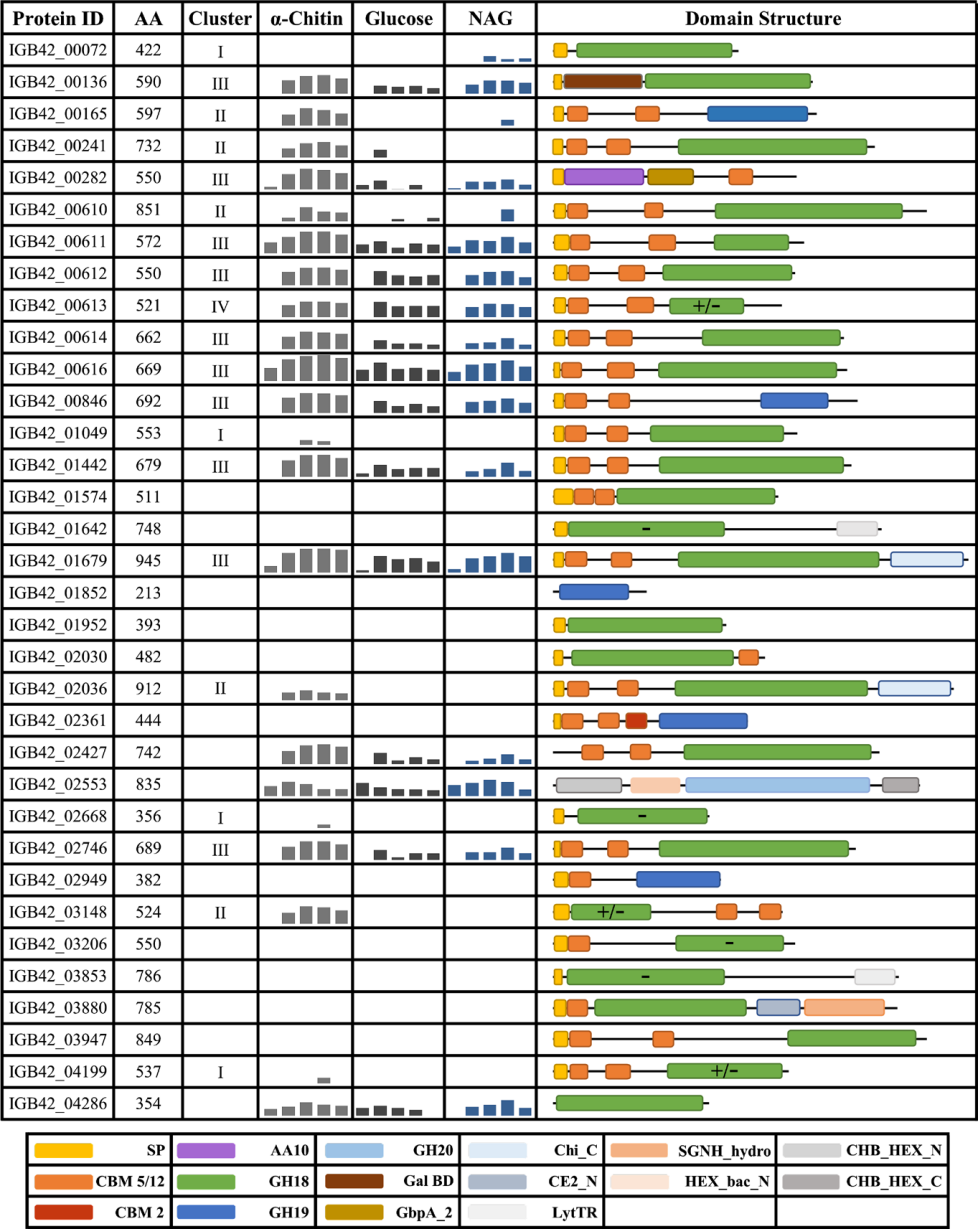
Putative chitin-active enzymes in the proteome of *A. ripae*. The figure shows all predicted *A. ripae* proteins containing domains annotated as glycosyl hydrolases in families GH18, GH19, or GH20 or as LPMO, with their domain architecture. The embedded bar charts show the average abundance during growth on α-chitin, glucose or *N*-acetylglucosamine for the five analyzed time points (1, 3, 5, 7, 13 days); the *y*-axis indicates protein abundance from 17 to 32 log2(LFQ); more detailed quantitative data is shown in Figure 2. The roman numbers in the column labeled “Cluster” refer to the clusters depicted in Figure 2. GH18 domains (green) that are predicted to lack catalytic activity are marked by “–”, whereas GH18 domains for which the prediction is uncertain are marked by “+/–”; see text for more details. Domains were annotated using InterProScan. HEX_bac_N: N-terminal domain of beta-hexosaminidases; GbpA_2: *N*-acetylglucosamine binding domain; CHB_HEX_N: N-terminal domain of chitobiases and beta-hexosaminidases, similar to CBM2/3, possibly involved in substrate binding; CHB_HEX_C: C-terminal domain of chitobiases and beta-hexosaminidases, no proposed catalytic or binding function; Gal_BD: Galactose binding domain; CE2_N: N-terminal domain of CE2 acetyl esterases; SGNH_hydro: SGNH hydrolase-type esterase domain with a similar fold to flavoproteins, often found in esterases and lipases; Chi_C: C-terminal domain found in some GH18s; LytTR: DNA-binding domain found in response regulators.

InterProScan analysis and manual curation revealed an exceptional number of enzymes that are (putatively) active on chitin. The analysis revealed 32 chitinases, of which 27 belong to the CAZy family GH18 and 5 belong to family GH19. For comparison, the corresponding numbers for the genomes of well-known chitinolytic bacteria such as *S. marcescens* and *C. japonicus* are four (4 GH18) and five (4 GH18, 1 GH19), respectively. In addition, the genome of *A. ripae* encodes one putative GH20 chitobiase and one AA10 LPMO. Figure 1 shows the predicted domain structures and the gene IDs for these 34 putatively chitinolytic enzymes, of which all but two GH18, one GH19, and the GH20 are predicted to be secreted. Of the 29 putative carbohydrate esterases, four belong to family CE4 and one to family CE14, i.e., families known to contain chitin deacetylases. There are examples of chitin degradation mechanisms that involve the action of chitin deacetylation and hydrolytic enzymes acting on deacetylated chitin oligomers.^32^ Chitosanases, occurring in CAZy families GH 46, 75, and 80, are likely not involved in the direct conversion of chitin but could be involved in hydrolysis of partially deacetylated chitin fragments. The *A. ripae* genome encodes only one putative chitosanase belonging to family GH46 (IGB42_01819).

Several of the proteins shown in Figure 1 contain at least one carbohydrate-binding module (CBM), which in most cases belong to the distantly related CBM5 and CBM12 families (referred to as CBM5/12) that are known to contain chitin-binding CBMs. In addition to these, one protein has a CBM2 along with the CBM5/12 pair, and some proteins have domains potentially binding to *N*-acetylglucosamine (GbpA_2 domain), galactose (Gal_BD domain), or cellulose (CHB_HEX_N). Interestingly, the genome of *A. ripae* encodes for 27 proteins that contain a CBM (either CBM5/12, CBM2, CBM50, or CBM66) but for which there is no other functional prediction that directly links them to chitin conversion. For some of these, InterProScan predicts other functions such as, e.g., peptidase activity. Several of these latter proteins were detected in the proteomics study and are discussed below.

### Expression of Chitinolytic Enzymes and Other CAZymes during Growth on Chitin

*A. ripae* was grown on plates containing 1% α-chitin, glucose or *N*-acetylglucosamine as the sole carbon source. Bacterial growth on the plates, above the filter, increased over time, as shown for *N*-acetylglucosamine in Figure S1. Secretomes were collected from the bottom of the plates, below the filter, at different time points and in triplicates (three plates per condition and time point). Proteins were analyzed by high resolution LC-MS/MS and quantified using the MaxLFQ algorithm,^23^ showing adequate reproducibility with Pearson correlations ranging from *R* = 0.22 for early time points to *R* = 0.97 for later time points, between the triplicates (Figure S2; most values are >0.7). In total 1225 proteins were identified (Table S1) of which 216 (18%) are putatively secreted. This fraction of 18% is only slightly higher than the fraction of the total proteome that is predicted to be secreted, which is 13%. Thus, in this case, the plate method did not lead to strong enrichment of secreted proteins. Previous studies using this plate method allowed harvesting of secretomes that were enriched for secreted proteins with cytosolic fractions down to 9–55% for various fungi^33^ and about 30% for *C. japonicus*.^17^ On the other hand, in a study of *S. marcescens*, the cytosolic fraction was 60%.^8^

**Figure 2:**
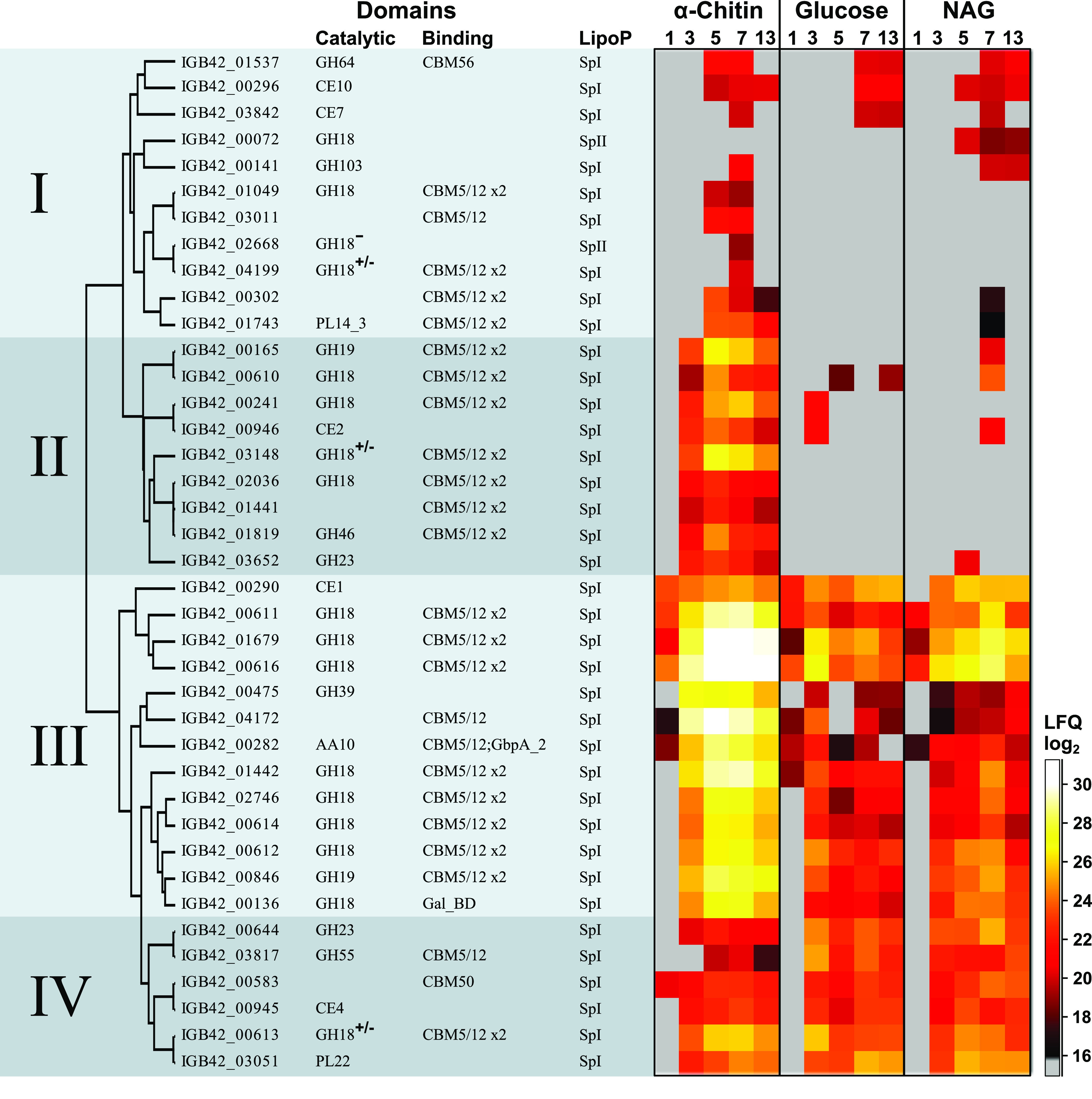
Heat map of secreted CAZymes. The figure shows a heat map of the 39 detected CAZymes that are predicted to be secreted for growth on α-chitin, glucose or *N*-acetylglucosamine, at five different time points (1–13 days). The color indicates the protein abundance, log2(LFQ), and represents the average of three biological replicates; gray color means not detected. The columns show protein ID’s, CAZy annotation for the catalytic and binding domains (auxiliary activity (AA), carbohydrate esterase (CE), glycosyl hydrolase (GH), polysaccharide lyase (PL)), the presence of carbohydrate-binding modules (CBMs), and the secretion pathway as predicted by LipoP. Superscripts at “GH18” indicate that this GH18 domain lacks (−) or possibly lacks (+/−) catalytic activity; see text for more details. The proteins were hierarchically clustered based on protein abundance patterns and manually divided into four groups as indicated: (I) Low expression on all three substrates; (II) Medium expression on α-chitin but not on glucose or *N*-acetylglucosamine; (III) High expression on all three substrates but clearly higher on α-chitin compared to glucose and *N*-acetylglucosamine; and (IV) Medium expression on all three substrates. GbpA_2: *N*-acetylglucosamine binding domain; Gal_BD: Galactose binding domain.

Looking closer at CAZymes, we detected 73 among the 1225 proteins, 39 of which are predicted to be secreted (Figure 2). A heat map of the 39 secreted CAZymes (Figure 2) revealed four clusters: Cluster I contains CAZymes with low and similar expression on all three substrates. Cluster II contains medium-abundant proteins that are upregulated during growth on α-chitin and that are not detected or show low abundance during growth on glucose or *N*-acetylglucosamine. Cluster III contains highly abundant proteins that are expressed with all three substrates but with clearly higher levels on α-chitin compared to glucose and *N*-acetylglucosamine. Cluster IV contains medium-abundant proteins expressed with all three substrates. Hence, proteins upregulated on chitin are found in clusters II and III and include 12 GH18s, 2 GH19s, the AA10 LPMO as well as three other GHs, two CEs and two proteins with a putative chitin-binding domain (CBM5/12) but no predicted catalytic activity. Note that the GH20 chitobiase is not visible in Figure 2 because it is predicted to be a cytosolic protein; Figure 1 shows that this protein (IGB42_02553) is equally abundant with all substrates and would thus fit to cluster IV. Of the three other nonsecreted chitinolytic proteins listed in Figure 1, IGB42_02427 (a GH18) showed the same expression pattern as proteins in Cluster III, IGB42_04286 (a GH18) showed the same expression pattern as the GH20, whereas IGB42_01852 (a GH19) was not detected.

It is clear that growth on chitin is associated with production of multiple chitin-active enzymes, including 20 of the 30 secreted chitinolytic proteins encoded in the genome (Table 1; 17 GH18, 2 GH19 + the AA10). Of these 20, 15 group in clusters II and III (12 GH18, 2 GH19, 1 AA10), meaning that they are clearly upregulated. Of the five secreted CAZymes in clusters II and III with no obvious chitinolytic activity, two are carbohydrate esterases, one belonging to a family known for its broad substrate specificity but lacking chitin deacetylases (CE1) and one belonging to a family of xylan esterases (CE2).^34^ Interestingly, two of the three glycoside hydrolases in this category, a GH23, a GH39, and a GH46, could be related to chitin conversion. The GH46 is a chitosanase that contains two CBM5/12s and that perhaps could act on partially deacetylated regions in the chitin substrate. The GH23 family contains a variety of peptidoglycan active enzymes some of which also show activity on chitin.

**Table 1:** Domain Structure, Detection, and Regulation of the Secreted Chitinases Listed in Figure 1^a^

	total	detected	upregulated
2 CBM 5/12	17 (14 GH18, 3 GH19)	14 (12 GH18, 2 GH19)	11 (9 GH18, 2 GH19)
2 CBM 5/12 + ChiC_N	2 (GH18)	2 (GH18)	2 (GH18)
1 CBM 5/12	4 (3 GH18, 1 GH19)	0	n.a.
LysM	2 (GH18)	0	n.a.
GalBD	1 (GH18)	1 (GH18)	1 (GH18)
no CBM	3 (GH18)	2 (GH18)	0
total	29 (25 GH18, 4 GH19)	19	14

a Proteins appearing in Clusters II or III in Figure 2 are defined as “upregulated”. Note that Figure 1 lists 34 chitinolytic proteins; four of these are not secreted and one is not a Chitinase but an LPMO. n.a., not applicable.

Cluster I, containing proteins of low abundancy with similar expression on all substrates, includes a polysaccharide lyase (PL14_3) with two CBM5/12s, two carbohydrate esterases (CE7, CE10), four GH18s, of which two carry two CBM5/12s, two glycoside hydrolases (GH64, GH103) and two proteins of unknown function carrying at least one CBM5/12. Cluster IV, containing the more abundant non-upregulated proteins contains one GH18 with two CBM5/12s, a putative chitin deacetylase (CE4), a PL22, a GH23, a GH55 [β-(1,3)-glucanases], and a protein of unknown function containing a CBM50 (known to bind to peptidoglycan and/or chitin). The two detected PLs, in clusters I and IV belong to families with enzymes acting on (alginate-related) glucuronan substrates. Although the enzymes in clusters I and IV do not seem upregulated during growth on chitin, several of them may still have functions related to chitin conversion, as suggested by the presence of CBM5/12 domains in several of these enzymes.

Table 1 provides an overview of the domain structures of the upregulated chitinases and reveals that chitinases with two CBM5/12 domains are overrepresented among the detected and upregulated enzymes. Such enzymes comprise 66% of the 29 secreted chitinases encoded in the genome, whereas they comprise 84% and 93% of the detected and upregulated secreted chitinases, respectively. On the other hand, none of the four secreted chitinases containing a single CBM5/12 nor the two secreted chitinases containing a LysM domain were detected. IGB_00136, which is a GH18 coupled to a putative galactose-binding domain, was detected and upregulated during growth on chitin (cluster III). Finally, two of the three chitinolytic proteins with no CBM were detected, but none of these were upregulated during growth on chitin. Although causal relationships cannot be derived from these observations, the overrepresentation of enzymes with two CBM5/12 domains in the genome and, more so, among the detected and upregulated proteins, suggests that chitinases with two CBMs are important members of the chitinolytic machinery of *A. ripae*. This is an intriguing observation since it is well-known that chitinolytic enzymes with less than two CBMs can be very effective. For example, all three chitinases from *S. marcescens*, generally considered to comprise an efficient chitinolytic machinery, have only one CBM.^9^ It remains to be studied how the presence of two CBMs, as opposed to only one CBM, affects Chitinase efficiency. It must be noted that the protein regions in between the CBMs and between CBMs and the GH18 domains vary in sequence and length (Figure 1) and that these linker regions likely need to be taken into account in future studies of the effect of CBMs on Chitinase efficiency.

GH18 catalytic domains carry several characteristic sequence motifs that are important for catalytic activity, as described in detail for ChiB from *S. marcescens*.^35^ These include the catalytically crucial D_140_XD_142_XE_144_ (numbering according to ChiB from *S. marcescens*) motif containing the catalytic acid/base (Glu144), the not crucial and not fully conserved S_93_XGG motif, and a Y_214_D/N_215_ motif that contains a tyrosine that plays a crucial role during catalysis.^35^ Sequence alignments showed that four of the 25 secreted GH18 proteins lack glutamate at position 144 in the D_140_XD_142_XE_144_ motif and thus likely lack catalytic activity. Only one of these was detected, in low amounts and without being upregulated (IGB42_02668; cluster I). The SXGG motif occurred in 21 of the 25 secreted GH18 proteins, including all detected proteins except IGB42_02688 (which also lacks parts of the DXDXE motif). Three more proteins, marked by “+/–” in Figures 1 and 2, may lack, or could have impaired, activity due to replacement of the conserved Tyr214 by methionine. This is uncertain, since the impact of a Tyr → Met mutation in this position has not been studied. All these three proteins were detected, and one of them (IGB42_03148) was upregulated during growth on chitin (the other two are IGB42_0613 in cluster IV and IGB42_04199 in cluster I). Importantly, this analysis strongly indicates that 13, and possibly 16, of the 17 detected GH18 proteins are catalytically competent. Of the 12 GH18 proteins that were clearly upregulated during growth on chitin, 11 seem catalytically competent, while this is less certain for one (IGB_03148).

It is worth noting that the upregulated GH18 chitinases include IGB42_00610, 00611, 00612, 00614, and 00616 that are encoded by adjacent genes. IGB42_00613 is also a secreted GH18 Chitinase, which was detected, but is possibly not active, and did not seem regulated (cluster IV in Figure 2). IGB42_00615 is annotated as a putative transcriptional regulator (not identified in the proteomic analysis) and is thus the only protein in this gene cluster with no obvious relation to chitin conversion. Analysis of the DNA sequence covering all the genes indicated above as well as the flanking regions by the Operon-Mapper software^36^ indicated that this gene cluster is not an operon. Nevertheless, the tight clustering of these genes on the genome may indicate co-regulation.

LPMOs are of major importance for efficient conversion of recalcitrant polysaccharides because they can act on crystalline regions that cannot be directly accessed by GHs.^13,37^ Indeed, the beneficial effect of LPMOs on enzymatic polysaccharide conversion is now well established, both in vivo,^38^ in vitro,^39^ and in industrial settings.^40,41^ The single LPMO encoded by the *A. ripae* genome (IGB42_00282, 550 residues) has not been functionally characterized, but both phylogenetic analysis of its catalytic domain and the fact that this enzyme was highly expressed during growth on chitin suggest activity on chitin.

**Figure 3:**
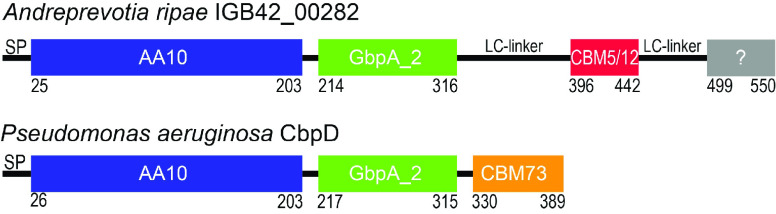
Domain structure of IGB_0282 and CbpD from *P. aeruginosa*. Domain boundaries are based on sequence analysis using InterPro. The gray box with a ? indicates a putative CBM with no current CAZy annotation, which is likely related to CBM5/12; see text for details. SP, signal peptide; LC-linker, low complexity region containing mainly Pro, Thr, Val, and Ala.

Interestingly, while the one LPMO of *S. marcescens*, known for its chitinolytic potential, is a single domain enzyme,^42^ IGB42_00282 contains three annotated domains, an LPMO domain, a GbpA_2 domain^43^ and a CBM5/12 (Figure 3). This domain organization is similar to that of CbpD, a 389 residue chitin-oxidizing virulence factor from *Pseudomonas aeruginosa*(44) (Figure 3). Furthermore, of all characterized chitin-active LPMOs, the catalytic domain of CbpD is the most similar to the catalytic domain of IGB_0282 (53% sequence identity). Still, the two enzymes show notable differences. Instead of the CBM5/12 domain in IGB42_00282, the CBM in CbpD is a CBM73. Furthermore, in IGB_0282, the CBM5/12 is linked to the GbpA_2 domain by a 60 residue long P- and T-rich linker, whereas the two domains are connected by a short (<10 residue) glycin-rich linker in CbpD. Moreover, in IGB42_00282, the CBM5/12 domain is followed by another long P- and T-rich linker and a domain with unknown function whose closest relatives are found in chitinases and lytic polysaccharide monooxygenases. This small (approx. 54 residues) domain contains multiple aromatic residues (3 Trp, 4 Tyr) and two cysteines and could very well be a chitin-binding domain. In support of this, the (unpublished) crystal structure of a family GH18 Chitinase from *Chromobacterium violaceum* (PDB ID 4TX8) shows an N-terminal domain, sharing 69% sequence identity with the unknown domain from IGB42_00282, that is positioned relative to the catalytic domain as one would expect for a chitin-binding domain (i.e., the surface of the putative CBM extends the substrate binding cleft in the catalytic domain, as seen for, for example, ChiB from *S. marcescens*(45)). In further support of a function in chitin-binding, this domain is annotated in InterPro as an IPR036573, which represents a superfamily of CBMs including CBM5 and CBM12.

**Figure 4:**
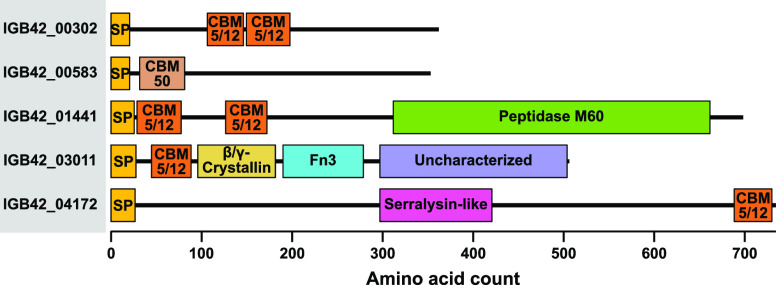
Detected secreted proteins with a putative chitin-binding domain but no known chitin-active catalytic domain. All these proteins, except IGB42_00583, were upregulated during growth on chitin (Figure 2). InterPro accession numbers for the non-CAZy domains are Peptidase M60: IPR031161; Beta/gamma Crystallin: IPR001064; Fibronectin type III (Fn3): IPR003961; Uncharacterized: IPR007541; Serralysin-like metalloprotease: IPR011049. See main text for more details. SP: signal peptide.

Figure 2 includes five proteins that harbor at least one putative chitin-binding domain (four with a CBM5/12 and one with a CBM50) but for which no CAZyme activity could be predicted. Figure 4 shows the predicted domain structures for these five proteins. The CBM50 containing protein (IGB42_00583) was abundant but not regulated (cluster IV). The other four, all containing at least one CBM5/12 were upregulated during growth on α-chitin and could thus be hitherto undescribed enzymes involved in chitin conversion. Two of these proteins, IGB42_302 and IGB_03011 appear in cluster I but do show increased expression during growth on chitin. The only recognizable feature of the former is the presence of two CBM5/12 domains. IGB42_03011 contains one CBM5/12 domain, one beta/gamma Crystallin domain, one Fibronectin type III like domain and one uncharacterized domain with similarity to basic secretory proteins found in plants that, according to InterPro, may be involved in defense against pathogens. Blast searches showed that homologues of IGB42_302 primarily occur in chitinolytic bacteria, whereas such searches did not reveal such an association for IGB42_03011.

IGB42_01441 appears in cluster II meaning that it is modestly expressed and clearly upregulated; it contains two CBM5/12 domains and one peptidase M60 domain that is believed to target complex glycoproteins such as mucus.^46^ Blast searches showed that the closest homologues of IGB42_01441 occur in other chitinolytic bacteria, adding to the notion that this protein may play a role in the degradation of chitinous material. Identification of chitin-binding proteases is not unexpected as chitin is commonly associated with structural proteins (e.g., insect cuticles^47^) that may need to be removed to provide access to chitin chains for the chitinolytic enzymes. IGB42_04172 occurs in cluster III and is of particular interest because it is one of the most abundant secreted proteins during growth on chitin. It contains one CBM5/12 domain and one serralysin-like metalloprotease C-terminal domain, but there is no predicted function for most of this 734 residue long protein. This serralysin domain, usually found in Zn-endopeptidases, is able to bind Ca^2+^-ions, and is believed to be involved in protein secretion.^48^ In addition to being associated with protein, most chitin-containing structures also contain substantial amounts of CaCO_3_, and it is conceivable that a Ca^2+^-binding domain has functions associated with chitin degradation. Close homologues of IGB42_04172 are found in a wide range of Gram-negative bacteria and are sometimes annotated as sugar-binding protein. These five proteins, and IGB42_00302, IGB42_01441, and IGB42_04172 in particular, may have hitherto undetected capabilities that are beneficial for chitin turnover and the identification of their functions is an interesting topic for future research.

### Expression of Other Proteins during Growth on Chitin

**Figure 5:**
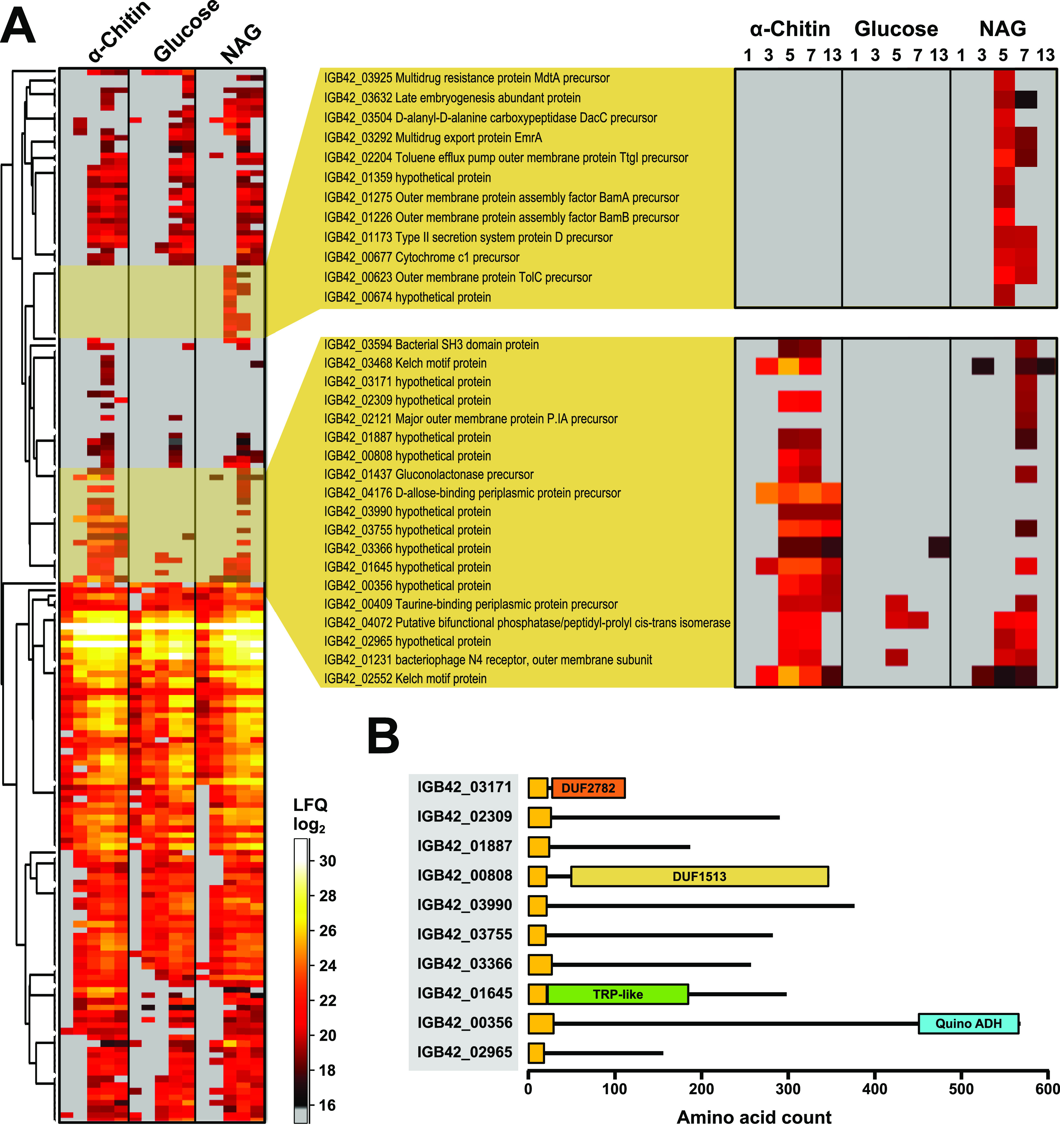
Heat map of secreted non-CAZymes. (A) The figure shows a heat map of the 177 detected non-CAZymes that are predicted to be secreted for three different substrates at five different time points (1–13 days, as shown in the zoomed regions). The color indicates protein abundance, log2(LFQ), and is based on the average of three biological replicates; gray color means not detected. The proteins were hierarchically clustered based on protein abundance patterns. NAG: *N*-acetylglucosamine. (B) Domain architecture of the 10 hypothetical proteins found in the lower of the two enlarged clusters, all upregulated on NAG and α-chitin. Signal peptides are shown in yellow. DUF: domain of unknown function; TPR: Tetratricopeptide (IPR011990); ADH: Alcohol dehydrogenase (IPR011047).

Of the 216 detected proteins that are predicted to be secreted, 39 are CAZymes, as discussed above. The remaining 177 proteins are also of interest, especially if they are upregulated during growth on chitin. Figure 5 shows that expression of the vast majority of secreted non-CAZyme proteins did not vary between the substrates. Thus, the clear response to chitin, described above, only concerns a subset of the secreted proteins, most of them with predicted chitinolytic activity. It is noteworthy that there is little difference between the secretome during growth on glucose and the secretome during growth on *N*-acetylglucosamine, which perhaps may be taken to support the notion that growth on the latter is “default” for *A. ripae*. Two clusters, highlighted in Figure 5, stand out, one containing proteins only detected for growth on *N*-acetylglucosamine and one with proteins detected for growth on both chitin and *N*-acetylglucosamine. Because these proteins generally were not very abundant and often not detected at all time points, their role in chitin conversion remains uncertain. Still, there are clear overall trends, and several of the proteins in the lower of the two highlighted clusters (Figure 5A) are both quite abundant and clearly upregulated. Most of these proteins are hypothetical proteins (Figure 5A,B). The two proteins with a Kelch motif (IGB_02552 and IGB_03468), which were detected in relatively large amounts in some of the chitin samples (Figure 5A), share 39% sequence identity and are predicted to contain a beta-propeller made up of multiple Kelch motifs, as in, e.g., galactose oxidase.^49^ Both proteins also contain BACON (Bacteroidetes-Associated Carbohydrate-binding Often N-terminal) domains that may be involved in sugar binding.^50^

BLAST searches of the 18 proteins in the lower enlarged cluster in Figure 5A showed that the closest homologues of many of these proteins, which are upregulated during growth on chitin, occur in genera such as *Chitinimonas*, *Chitiniphilus*, *Chitinibacter*, and *Chitinilyticum*, which all, like *A. ripae*, belong to the *Chromobacteriaceae* family. A possible role of these proteins in the utilization of chitin remains to be established and the expression data discussed above indicate that they are less dominant in the proteome than the (predicted) true chitinases.

Assessment of detected CAZymes that are not predicted to be secreted did not reveal any proteins that were upregulated during growth on chitin, with the exception of IGB42_02427, which is a GH18 with two CBM5/12 domains that lacks a secretion signal (Figure 1).

Taken together, the genomic and proteomic data described here show that *A. ripae* has an extraordinarily large chitinolytic machinery and that a large part of this machinery is indeed put to action during growth on chitin. The clearly upregulated proteins are strongly dominated by GH18 enzymes containing two CBM5/12 domains, whereas the LPMO and a CBM5/12-containing protein of unknown function (IGB42_04172) are also abundantly expressed. It may seem as if *A. ripae* is predisposed to live in a chitin-rich niche, since there is considerable expression of chitinases even under conditions where chitinases do not seem necessary, i.e., growth on glucose or *N*-acetylglucosamine (e.g., Cluster III in Figure 2). The present observations indicate that regulatory mechanisms in *A. ripae* differ from those in, e.g., the well-known chitin degrading bacterium *S. marcescens*, for which previous proteomic studies revealed a highly specific response to chitin.^8^ In this latter case, secreted chitin-active enzymes were strongly upregulated in the secretome during growth on chitin, compared to growth on glucose.

As alluded to above, the large multiplicity of chitinases in *A. ripae* is rare in bacteria (for another example, see ref (51)) and resembles the multiplicity found in certain fungi (e.g.,^52^). It remains to be seen if the many different proteins are used to degrade chitin more efficiently or whether they reflect an ability to degrade a wider variety of chitin-containing (copolymeric) substrates, for example, including both chitin from the insect and from insect-associated fungi.^53^ It is also conceivable that the GH18 enzymes have different temperature and pH optima, allowing the bacterium to degrade chitin under varying conditions.^54^ Functional characterization of all the 29 chitinases, or of the 14 that were both detected and upregulated, could provide insight into the unique catalytic machinery of *A. ripae*. Notably, this will be a massive task, because several of these multidomain enzymes are likely difficult to express and assessment of synergistic effects between the many enzymes would be quite demanding. Furthermore, while chitinase activity can easily be verified with artificial substrates, assessment of essential functional properties related to chitin-processing, such as exo- vs endoaction and processivity and its directionality, is highly challenging (e.g., refs (9, 55−57)). It is conceivable that expression and functional characterization of individual chitinases produced by *A. ripae* will lead to discovery of useful biocatalysts.

Since chitin is often associated with proteins and other polysaccharides, it is conceivable that other, hitherto not characterized enzymes co-determine the efficiency of the degradation of chitin-rich biomass. Indeed, we found several proteins with no known chitinolytic function that are predicted to be secreted and that were upregulated during growth on chitin (Figures 4 and 5). These proteins are interesting targets for further work aimed at unravelling on how nature converts chitin-rich materials. The five proteins listed in Figure 4 are of special interest, since the presence of chitin-binding domains suggests a role in chitin conversion.

Next to shedding new light on natural conversion of chitin-rich materials, the present study reveals a reservoir of novel enzymes that may find applications in industrial processing of chitin-rich biomass. Despite the abundance of such biomass, e.g., crustaceans or farmed insects, it remains challenging to develop green methods for extracting the chitin^2,5,58^ and to develop efficient enzyme technologies for chitin valorization.^59^ Enzymes produced by *A. ripae* may be explored in the development of novel methods for chitin extraction, and the best chitinases could, for example, be used to efficiently convert chitin to monosugars, for further valorization by fermentation, or to chito-oligomers with interesting bioactivities.

## Supporting Information Available

Figure S1: Pictures of *A. ripae* growing on plates; Figure S2: Multiscatterplots of protein abundances to illustrate reproducibility of protein quantification (PDF)
Table S1: Data for all detected proteins (XLSX)
