# Supplementary material for: Genomic and Proteomic Study of Andreprevotia ripae Isolated from an Anthill Reveals an Extensive Repertoire of Chitinolytic Enzymes
Source: J Proteome Res. 2021 Jun 30;20(8):4041–52. doi: 10.1021/acs.jproteome.1c00358 (PMC8802321; doi:10.1021/acs.jproteome.1c00358)
Supplement: Supplementary file 1 — pr1c00358_si_001.pdf [file pr1c00358_si_001.pdf]

Genomic and proteomic study of *Andreprevotia ripae* isolated from an anthill reveals an extensive repertoire of chitinolytic enzymes

Silje B. Lorentzen[1], Magnus Ø. Arntzen[1], Thomas Hahn[2], Tina R. Tuveng[1], Morten Sørlie[1], Susanne Zibek[2], Gustav Vaaje-Kolstad[1], Vincent G. H. Eijsink[1*]

[1] Faculty of Chemistry, Biotechnology and Food Science, NMBU - Norwegian University of Life Sciences, N-1433 Ås, Norway

[2] Fraunhofer Institute for Interfacial Engineering and Biotechnology IGB, Nobelstraße 12, 70569 Stuttgart, Germany

*For correspondence; e-mail: vincent.eijsink@nmbu.no

Supplementary Information

Contents:

Figure S1. Growth of *A. ripae* on plates.

Figure S2. Multi-scatterplots of protein abundances.

Table S1. All detected proteins (separate Excel file).

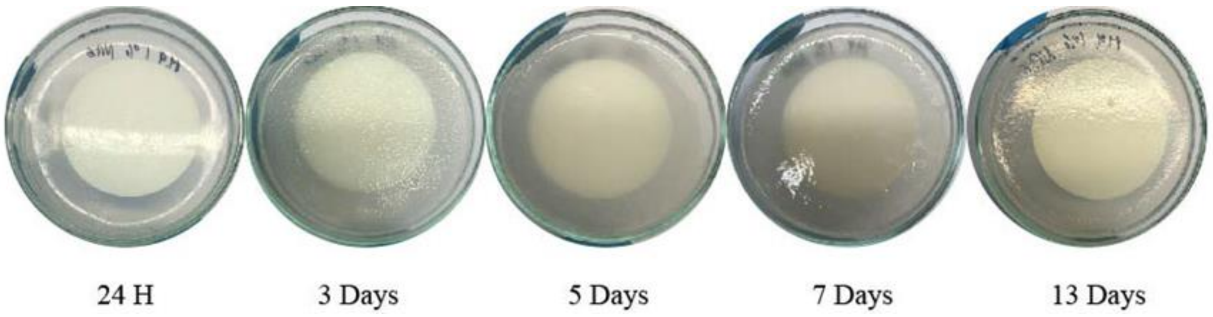

Figure S1. Growth of *A. ripae* on plates. The figure shows growth of *A. ripae* on plates containing *N*-acetylglucosamine as carbon source for the time course of the study. The increase in the number of bacterial cells over time results in increased surface turbidity.

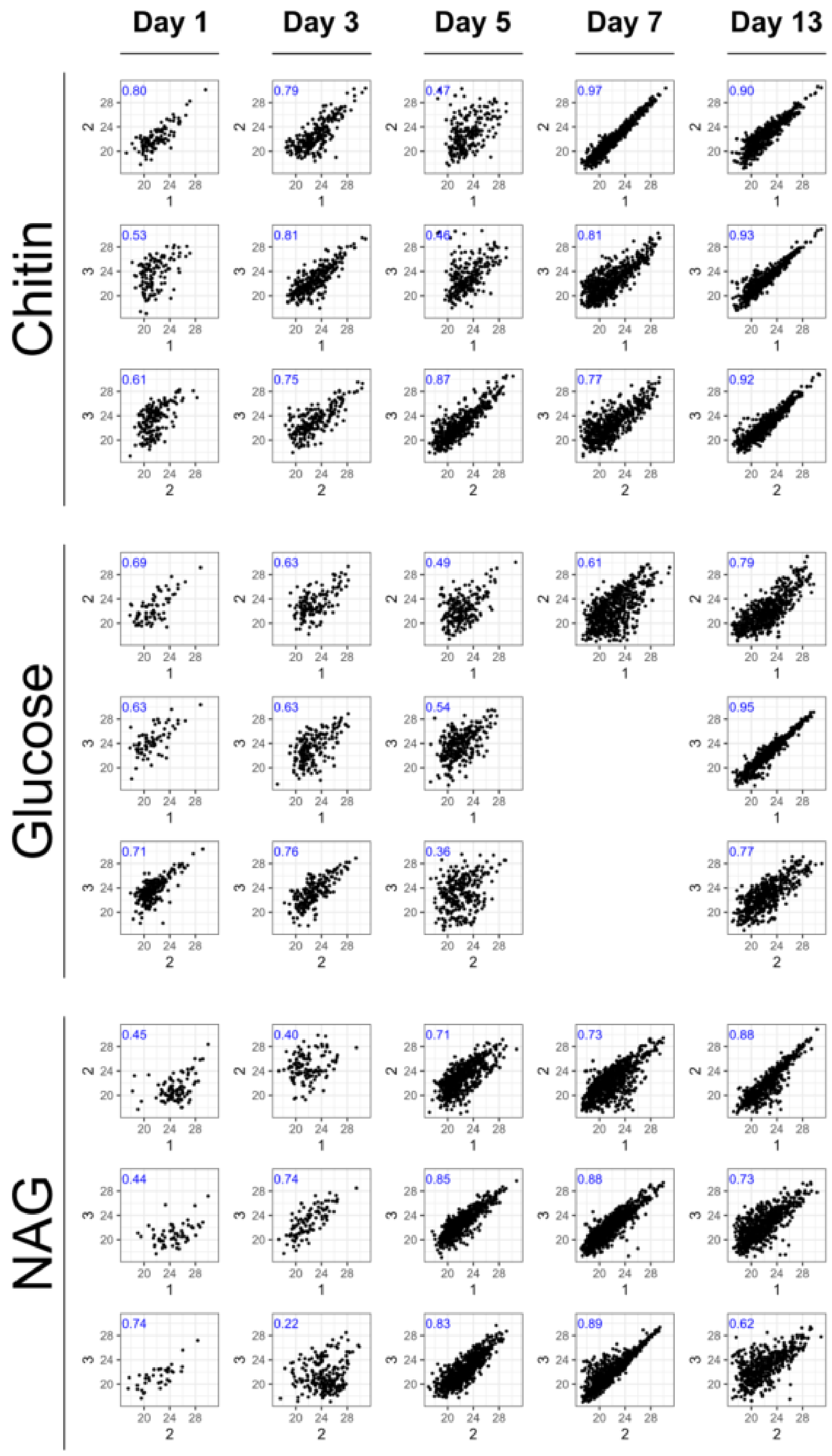

Figure S2. Multi-scatterplots of protein abundances. Three biological replicates for each condition and time point were pair-wise plotted against each other to illustrate the reproducibility of quantification. Blue numbers indicate Pearson correlations and range from R=0.22-0.97. Expectedly, samples from early time

points contain fewer secreted proteins and show lower correlation between replicates. For growth on glucose at day 7, only two replicates were available hence only one scatterplot could be made. Both axes are protein abundances, log2(LFQ), and labels indicate the replicate number. NAG: *N*-acetylglucosamine.

Table S1. All detected proteins. The Table (Excel file) provides information, including proteomics quantification, for the in total 1225 proteins that were detected in the secretome of *A. ripae* when grown on α-chitin, glucose or *N*-acetylglucosamine.
